# Prevalence and associated factors of medication adherence among infertile women undergoing frozen-thawed embryo transfer cycle: A cross-sectional study

**DOI:** 10.3389/fphar.2023.1148867

**Published:** 2023-03-17

**Authors:** Ying Ni, Chenye Tong, Lianying Xu, Wen Qian, Limin Huang, Aijun Zhang, Qiong Fang

**Affiliations:** ^1^ Department of Gynecology and Obstetrics, Ruijin Hospital, Shanghai Jiao Tong University School of Medicine, Shanghai, China; ^2^ Department of Nursing, Ruijin Hospital, Shanghai Jiao Tong University School of Medicine, Shanghai, China; ^3^ School of Nursing, Shanghai Jiao Tong University, Shanghai, China

**Keywords:** frozen-thawed embryo transfer (FET), associated factors, medication adherence, infertile women, self-efficacy

## Abstract

**Objective:** This study aimed to explore the prevalence and associated factors of medication adherence among infertile women undergoing frozen-thawed embryo transfer (FET) cycle.

**Methods:** A cross-sectional study was conducted with 556 infertile women undergoing FET cycle in total. The Self-efficacy for Appropriate Medication Use Scale (SEAMS), Herth Hope Index (HHI) scale, and Social Support Rating Scale (SSRS) were used to evaluate the patients. Data were described by univariate and multivariate analyses. Logistic regression method was performed to analyse the factors potentially associated with medication adherence.

**Results:** The average score of Self-efficacy for Appropriate Medication Use Scale (SEAMS) was 30.38 ± 6.65, and 65.3% of participants showed non-adherence. Multiple regression analysis indicated that first-time FET cycle, treatment stage, methods of daily medication, social support and hope level were the main associated factors of the medication adherence among infertile women undergoing FET cycle (*p* < 0.001).

**Conclusion:** This study revealed the medication adherence is at medium level among infertile women undergoing FET cycle, especially in patients with repeated FET cycles. The study also suggested that improving the hope level and social support of infertile women undergoing FET cycle may increase medication adherence.

## Introduction

Infertility is a substantial healthcare and social problem. According to the statistics from the World Health Organization (WHO), approximately 8%–12% (Schmidt et al., 2005) of couples in the world have suffered from infertility. With the development of human assisted reproductive technology, *in vitro* fertilization/intracytoplasmic sperm injection embryo transfer (IVF/ICSI-ET) has become the main treatment method for infertility, and has brought hope to many infertile couples (Dunn et al., 2014). Embryo transfer includes fresh embryo transfer (ET) and frozen-thawed embryo transfer (FET). Over the past decade, FET has increased significantly with the expansion of indications for this procedure, partly due to the improvements of vitrification compared with the older slow-freeze methods (Rienzi et al., 2017). In the United States, embryo cryopreservation with subsequent FET has increased from 7.9% of cycles in 2004% to 40.7% in 2013 (Christianson et al., 2017), with a similar increase worldwide (Casper and Yanushpolsky, 2016; Adamson et al., 2018; Division of Reproductive HealthNational Center for Chronic Disease Prevention and Health Promotion, 2018). In addition, in recent years, the use of the freeze-only strategy for cryopreservation of all potentially viable embryos has increased steadily (Shapiro et al., 2014; Roque et al., 2019).

Medication adherence can be defined as the extent to which a patient’s behavior conforms to a prescribed medication dosing regime, including timing, dosing and medication intake interval (Cramer et al., 2008; Vrijens et al., 2012). High medication adherence can improve drug efficacy and enhance disease prognosis. It has become a decisive factor affecting the efficacy, especially when the treatment regimen is effective (Zhang et al., 2006). Improving medication adherence may have a greater influence on people’s health than any new medical discovery (Brown et al., 2016). A previous study showed that about a quarter of patients were non-adherent, with patients in acute conditions having a higher rate of adherence than those in chronic conditions (DiMatteo, 2004). Even in the resource-intensive setting of clinical trials, the average adherence rates for investigational drugs for chronic diseases just ranged from 43 to 78 percent (Waeber et al., 1999; Claxton et al., 2001; Cramer et al., 2003).

Infertility treatment regimens are often complex and can impede a woman’s daily life (Boivin et al., 2012). The treatment cycle includes oral and/or injection routes. The medication schedule varies from daily to multiple doses per day, and is basically very sensitive to time (Smith et al., 2003). Failure to take medications correctly during the treatment cycle decreases the likelihood that the medications will have the intended effect on reproductive hormones and ovarian follicle development, ultimately increasing the medical cost of repeat treatments (Noorhasan et al., 2008; Katz et al., 2011). So far, assessment of medication adherence in infertile women have not been widely researched, let alone in those undergoing FET cycle. This may be related to people’s traditional sense that those patients will definitely have high adherence because of their overwhelming desire to have pregnancy. However, one research by Guo et al. (2020) revealed that only 30.4% of infertile women had high medication adherence undergoing IVF treatment, which was lower than that of patients with hypertension and other diseases (Huang et al., 2018; Pinho et al., 2021; Zhou et al., 2022). The patients undergoing FET cycle normally have a long treatment duration, and often meet the following situations: complicated medication regimen, prescribed dosage frequency, adverse drug reactions and other factors, which have caused great trouble to them. And these factors have also been proved to significantly impact the medication adherence of patients with primary infertility (Kruse et al., 1991; Kruse et al., 1993).

Moreover, the patients usually take drugs at home and could not get the guide from the medical staff in time. Meanwhile, we conducted a cross-sectional survey in our previous study, which indicated that the infertile women undergoing repeated IVF cycles have less social support and lower hope levels (Ni et al., 2021). These psychosocial aspects might also have an influence on adherence (Gu et al., 2017; Seki Öz et al., 2022a). Social support is the “available external resources” for individuals when facing stress (Zhong et al., 2019). It is usually defined as the perceived comfort, care, help and respect that a person obtains from others (Wallston et al., 1983). It can help individuals reduce perceived stress, lessen the impact of negative emotions, and improve self-efficacy. Recent studies showed that social support presented a positive effect on medication adherence on various diseases (Gu et al., 2017; Hacihasanoglu Asilar et al., 2020). Hope is a multi-dimensional positive power. It provides an optimistic expectation for good outcomes and an effective adjustment mechanism that enables individuals to overcome current difficulties (Yadav, 2010). Hope is a factor that initiates and sustains the healing process. It is also an important coping mechanism that facilitates fighting diseases and improves insight and treatment adherence (Seki Öz et al., 2022b). Hope has been found to be associated with adherence in seropositive individuals in a methadone program, and individuals with spinal cord injury, relapsing-remitting multiple sclerosis, schizophrenia, and women with breast cancer (Fraser et al., 2001; Seki Öz et al., 2022a).

These mentioned factors may affect the medication adherence in infertile women undergoing FET cycle, and then affect the final pregnancy outcome. Therefore, the purpose of this study is to investigate the prevalence of medication adherence among infertile women undergoing FET cycle, and explore its possible associated factors, so as to provide basis for taking appropriate interventions to improve patients’ adherence and in tune the treatment outcome.

## Materials and methods

### Data and study design

An observational, cross-sectional study with conveniet sampling was conducted at the Reproductive Medical Center of Ruijin Hospital affiliated to Shanghai Jiao Tong University School of Medicine. All participants were recruited among infertile women undergoing FET cycle from May to December 2021. The inclusion criteria were as follows: Infertile women undergoing FET, aged between 20 and 45, agree to participate in the study and have the ability to complete the survey. The investigation was conducted in a random follow-up period within a FET cycle. The study protocol was in accordance with the ethical standards and was approved by the Ethics Committee of Shanghai Ruijin Hospital (2020LLSD-158). After obtaining the written informed consent of this study, a self-reported questionnaire was distributed to each eligible participant, and clinical data were collected from their medical records.

The sample size was estimated by the formula: n = Z_1-α/2_^2^ * P*(1-P)/d^2^ (Charan and Biswas, 2013) with 95% of confidence interval. According to a published study (Guo et al., 2020), we selected 65% as the adherence rate within patients undergoing FET, and 0.05 for the assuming precision. It was estimated that 350 participants would meet the requirement based on the sample size calculation. Considering the 20% dropout rates, the minimum sample size was 438 participants.

In total, 600 eligible participants were recruited, of which 29 patients declined to participate, and 15 questionnaires were excluded due to missing answers or identical answers to each question. A total of 556 complete responses were received, with an effective rate of 92.7%.

### Measures

The demographic characteristics and clinical information were retrieved from a general information questionnaire and the medical records, including age, occupation, education level, monthly income, attribution of infertility, types of infertility, duration of infertility, number of attempted FET cycles, daily medication methods and daily drugs categories, etc.

The self-efficacy for appropriate medication use scale (SEAMS) was used to assess the medication adherence. Self-efficacy, defined as the belief or confidence that one can successfully perform a specific action required to attain a desired outcome, has been found to be an important predictor of medication adherence (Risser et al., 2007). Based on this concept, Risser et al. (2007) developed the SEAMS to incorporate the evaluation of self-efficacy in assessing medication adherence. The scale is now widely used to measure the medication adherence in various fields including hypertension, heart disease, diabetes and stroke, and has been proven to have good reliability and validity (Dong et al., 2015; Chen and Chen, 2017; Alhazzani et al., 2021). In prior to this study, we have verified the SEAMS in assessing the medication adherence among infertile women undergoing IVF-ET (Tong et al., 2020) with a good reliability. The SEAMS is a 13-item scale, with each item scored by Likert 3-level (1, not confident; 2, somewhat confident; and 3, very confident). The total score is 13–39. Higher score of each dimension and the overall level indicates higher medication adherence. Scores ≤26 are considered as low adherence, scores of 26–35 as moderate adherence, and scores ≥35 as high adherence (Tong et al., 2020). The Cronbach’s alpha coefficient of the SEAMS was 0.884 in this study.

The hope level was assessed using the Herth hope index (HHI) scale (Herth, 1992) in this study. The scale is widely used and has been proven to have good reliability and validity (Ni et al., 2021; Harmanci and Budak, 2022). The HHI scale consists of three dimensions: attitude towards reality and future, positive action, and keep interconnectedness with others. All items were scored with Likert 4-level scoring method, with 1-4 points representing very opposed, opposed, agreed and very agreed respectively. The total score of the scale is 12–48 points, of which 12–23 points, 24–35 points and 36–48 points representing low, medium and high levels of hope respectively. The higher the score, the higher level of hope of the respondents. The Cronbach’s reliability value of the scale was 0.85.

The social support was assessed by the Chinese version of the Social Support Rating Scale (SSRS) designed by Shuiyuan (1994). The scale has shown sufficient reliability and validity (Shen and Xing, 2020; Ni et al., 2021). There are 10 items in total, including objective support, subjective support and support utilization. The total score ranges from 12 to 66 points. Higher scores indicate more social support. In the present study, the Cronbach’s alpha coefficient of the SSRS Scale was 0.92.

### Statistical analysis

SPSS version 23.0 was used for statistical analysis in this study. The measured data was expressed as the mean ± standard deviation, and the count data was presented as frequency and constituent ratio (%). Student t-test was used to compare the two groups. The x^2^ test was used to check the rate inspection. Pearson correlation test was used to analyze the correlation between social support, hope, and medication adherence in infertile women undergoing FET cycle. The factors which were potentially associated with medication adherence were analyzed by multiple logistics regression analysis. *p* < 0.05 was considered statistically significant.

## Results

### Basic characteristics

In total, 556 infertile women undergoing FET cycle were included. The average age of participants was 32.68 ± 3.63 years, ranging from 21 to 45 years. The duration of infertility ranged from 1 to 18 years, with an average of 4.12 ± 1.67 years. Characteristics of included participants are summarized in Table 1.

**TABLE 1:** Participants’ characteristics by level of medication adherence.

Characteristics	Total n (%)	Low Adherence n (%)	Moderate Adherence n (%)	High Adherence n (%)	*χ2*	P
N	556	201 (36.2)	162 (29.1)	193 (34.7)		
First-time FET Cycle						
Yes	359 (64.6)	107 (29.8)	111 (30.9)	141 (39.3)	18.471	**<0.001**
No	197 (35.4)	94 (47.7)	51 (25.9)	52 (26.4)		
Treatment Stage						
Before embryo transfer	298 (53.6)	118 (39.6)	74 (24.8)	106 (35.6)	6.330	**0.042**
After embryo transfer	258 (46.4)	83 (32.2)	88 (34.1)	87 (33.7)		
Age (years)						
21-25	31 (5.6)	13 (41.9)	12 (38.7)	6 (19.4)	10.251	0.248
26-30	198 (35.6)	75 (37.9)	59 (29.8)	64 (32.3)		
31-35	206 (37.1)	69 (33.5)	54 (26.2)	83 (40.3)		
36-40	106 (19.1)	36 (34.0)	32 (30.2)	38 (35.8)		
41-45	15 (2.7)	8 (53.3)	5 (33.3)	2 (13.3)		
Duration of infertility (years)						
1-3	270 (48.6)	87 (32.2)	82 (30.4)	101 (37.4)	5.505	0.239
4-6	186 (33.5)	79 (42.5)	48 (25.8)	59 (31.7)		
≥7	100 (18.0)	35 (35.0)	32 (32.0)	33 (33.0)		
BMI index						
BMI<18.5	35 (6.3)	11 (31.4)	13 (37.1)	11 (31.4)	4.614	0.594
18.5≤BMI<24	352 (63.3)	137 (38.9)	98 (27.8)	117 (33.2)		
24≤BMI<28	118 (21.2)	35 (29.7)	37 (31.4)	46 (39.0)		
BMI≥28	51 (9.2)	18 (35.3)	14 (27.5)	19 (37.3)		
Educational level						
high school or below	123 (22.1)	62 (50.4)	33 (26.8)	28 (22.8)	23.076	**0.001**
Senior high/Technical secondary school	105 (18.9)	44 (41.9)	31 (29.5)	30 (28.6)		
Junior College/Undergraduate	285 (51.3)	82 (28.8)	86 (30.2)	117 (41.1)		
Master’s degree or above	43 (7.7)	13 (30.2)	12 (27.9)	18 (41.9)		
Occupation, n (%)						
physical	39 (7.0)	20 (51.3)	11 (28.2)	8 (20.5)	12.715	0.122
mental	397 (71.4)	134 (33.8)	118 (29.7)	145 (36.5)		
unemployed	120 (21.6)	47 (39.2)	33 (27.5)	40 (33.3)		
Family monthly income (Yuan)						
≤5000	240 (43.2)	100 (41.7)	67 (27.9)	73 (30.4)	6.123	0.190
5001∼10000	160 (28.8)	51 (31.9)	47 (29.4)	62 (38.8)		
>10000	156 (28.1)	50 (32.1)	48 (30.8)	58 (37.2)		
Self-assessment of financial difficulties						
no difficulty	236 (42.4)	70 (29.7)	66 (28.0)	100 (42.4)	13.640	**0.009**
slight difficulty	252 (45.3)	107 (42.5)	76 (30.2)	69 (27.4)		
great difficulty	68 (12.2)	24 (35.3)	20 (29.4)	24 (35.3)		
Types of daily medication						
<3	117 (21.0)	36 (30.8)	28 (23.9)	53 (45.3)	14.437	**0.006**
3-5	297 (53.4)	101 (34.0)	90 (30.3)	106 (35.7)		
>5	142 (25.5)	64 (45.1)	44 (31.0)	34 (23.9)		
Methods of daily medication						
<3	361 (64.9)	116 (32.1)	96 (26.6)	149 (41.3)	19.652	**<0.001**
≥3	195 (35.1)	85 (43.6)	66 (33.8)	44 (22.6)		
Type of infertility						
primary infertility	328 (59.0)	121 (36.9)	94 (28.7)	113 (34.5)	0.199	0.905
secondary infertility	228 (41.0)	80 (35.1)	68 (29.8)	80 (35.1)		
Attribution of infertility						
female factors	337 (60.6)	106 (31.5)	98 (29.1)	133 (39.5)	13.652	**0.008**
male factors	79 (14.2)	33 (41.8)	19 (24.1)	27 (34.2)		
bilateral factors	140 (25.2)	62 (44.3)	45 (32.1)	33 (23.6)		

## Participants’ medication adherence

Overall, the total score of SEAMS was 30.38 ± 6.65, which showed a medium level of medication adherence among infertile women undergoing FET cycle. Only 34.7% (193/556) participants showed high adherence (37.55 ± 1.62), 29.1% (162/556) moderate adherence (31.01 ± 2.39), and 36.2% (201/556) low adherence (22.98 ± 3.21). See Table 2 for the score and percentage of each part.

**TABLE 2:** Medication adherence among infertile women undergoing FET cycle.

Categories	Mean ± SD	No. of cases	Percentage (%)
Low adherence	22.98 ± 3.21	201	36.2
Medium adherence	31.01 ± 2.39	162	29.1
High adherence	37.55 ± 1.62	193	34.7
SEAMS Total	30.38 ± 6.65	556	100

### Univariate analysis

The medication adherence among infertile women undergoing FET cycle differed in the following factors: first-time FET cycle, treatment stage, educational level, self-assessment of financial difficulties, types of daily medication, methods of daily medication, and attribution of infertility (*p* < 0.05). See Table 1 for details.

### Correlation analysis

Pearson correlation analysis showed that medication adherence was positively correlated with hope level (HHI) (r = 0.542, *p* < 0.001) and social support (SSRS) (r = 0.600, *p* < 0.001), as shown in Table 3. It was indicated that the patients with higher hope level and more social support would have higher medication adherence.

**TABLE 3:** Correlation analysis between SEAMS and HHI & SSRS.

Item	Mean ± SD	*r*	P
HHI Total Score	36.83 ± 4.68	0.542	0.007
T^1^	12.44 ± 1.70	0.500	< 0.001
P^2^	12.35 ± 1.64	0.486	0.008
I^3^	12.03 ± 1.88	0.475	0.001
SSRS Total Score	39.89 ± 6.54	0.600	0.004
objective support	9.55 ± 2.49	0.477	0.007
subjective support	22.37 ± 3.64	0.524	< 0.001
support utilization	7.907 ± 1.84	0.447	0.002

1) Attitude towards reality and future. 2) Positive action. 3) Keep interconnectedness with others.

### Factors associated with medication adherence

The medication adherence result (Low adherence = 1, Moderate adherence = 2, High adherence = 3) was regarded as a dependent variable. The variables with statistical significance in univariate analysis and the HHI & SSRS scores were included in the multivariate logistic regression model. The results of multivariate logistic regression analysis showed that first-time FET cycle, treatment stage, methods of daily medication, total score of SSRS and HHI were the main factors which were potentially associated with the medication adherence among infertile women undergoing FET cycle (*p* < 0.01), as shown in Table 4.

**TABLE 4:** Logistic regression analysis of factors associated with medication adherence among infertile women undergoing FET cycle.

	Variable	β	SE	*χ2*	df	P	OR (95%CI)
SSRS Score	/	0.172	0.018	89.352	1	<0.001	1.19 (1.15–1.23)
HHI Score	/	0.236	0.026	82.193	1	<0.001	1.27 (1.20–1.33)
First-time FET cycle	No	-					
	Yes	0.908	0.204	19.827	1	<0.001	2.48 (1.66–3.70)
Stage of embryo transfer	after	-					
	before	−0.507	0.221	5.267	1	0.022	0.60 (0.39–0.93)
Methods of daily medication	≥3	-					
	<3	0.622	0.287	4.691	1	0.030	1.86 (1.06–3.27)

Only statistically significant coefficients (B) with 95% CIs are demonstrated here.

## Discussion

### Medication adherence among infertile women undergoing FET cycle

Medication adherence is a crucial point for the success and safety of many therapies (Bitton et al., 2013; Kim et al., 2018; Martin-Ruiz et al., 2018). Medication non-adherence is a widespread problem, which causes high costs globally (Burkhart and Sabaté, 2003; Bitton et al., 2013; Cutler et al., 2018). Especially in chronic conditions with long-term therapies, adherence is very important to achieve target outcomes, but it is usually low (Burkhart and Sabaté, 2003). This study showed that the high medication adherence rate among infertile women undergoing FET cycle was only 34.7%, which was similar to the results of Guo et al. (2020). The result indicated that the medication adherence among infertile women undergoing FET cycle remains a medium level. This is slightly lower than the 50.0% adherence rate among infertile women with endometriosis undergoing Marvelon treatment investigated by Xu et al. (2011), which may be related to the more complex treatment regimen of FET cycle.

The patients undergoing FET cycle normally have a long treatment duration, and some patients with endometrial factors may even endure three to 4 months. Those patients need to take several different drugs at different treatment stage in one FET cycle, and sometimes have to adjust medication regimen based on the subsequent conditions during the treatment. In some cases, the patients have even three to five medication methods (including oral administration, vaginal obstruction, subcutaneous injection, etc.) daily. Moreover, the patients usually take drugs at home and could not get the guide from the medical staff in time. In general, all these factors require patients to have good understanding and high self-efficacy.

### Analysis of associated factors of medication adherence

Our study indicated that first-time FET cycle, treatment stage, methods of daily medication, social support and hope level were the main factors which were associated with the medication adherence among infertile women undergoing FET cycle. The result of logistic regression analysis showed that the patients in first-time FET cycle were significantly more adherent than those in repeated cycles. This might because the patients in first-time FET cycle had great believes in the treatment plans and high expectations for the successful outcome. However, once the first attempt fails, psychological stress and disappointment will increase significantly, leading to a significant decline in their level of medication adherence. This suggests that medical staffs should pay more attention to those in repeated FET cycles, offer psychological counselling according to their psychological status, and enhance their self-confidence and belief in medication regimen.

Patients with less (<3) methods of daily medication showed higher medication adherence. This result indicated complicated medication regimen is negatively correlated with medication adherence, which is consistent with the research by Kruse et al. (1993). From a practical point of view, it seems reasonable to use the simplest possible treatment regimens, provided that they are appropriate in pharmacology and clinical.

This study also found that patients before embryo transfer in FET cycle were more likely to be lower adherence. This may because some of the patients need early surgical intervention or medication due to endometrial factors (intrauterine adhesion, adenomyosis, endometriosis, multiple hysteromyoma, etc.) before embryo transfer. Variable drugs regimens, together with complex medication methods, make it difficult for patients to remember all medications and easy to be confused. These factors easily lead to poor medication adherence. However, the medication regimen is basically fixed and normally with only one method of oral administration after embryo transfer, which results in higher medication adherence. This suggests that medical staffs should pay more attention to patients before embryo transfer. According to the literature report (LINN et al., 2018), the multivariate intervention of clinical education, medication card and combined strategies can generally improve the medication adherence. So, it is suggested to conduct multivariate behavioral interventions, to get an improved medication adherence.

The score of hope level in this research was at high level and consisted with the result by TANG et al. (2018). Pearson correlation analysis of hope level and medication adherence showed that there was a positive correlation between them. Those who have higher hope level are more likely to have better adjustment of psychological adaptation, more likely to seek medical and family support, and more willing to cooperate with clinical treatment. Therefore, the patients with higher hope level have higher medication adherence. Our previous study found that the hope level of the patients of first-time IVF cycle was significantly higher than that of the repeated cycle (Ni et al., 2021). This also explained the result in this study, that is, the patients in first-time FET cycle were significantly more adherent than those in repeated cycles. So, it is suggested to improve the hope level of patients to improve their medication adherence. Nurses need to provide appropriate guidance, suggestions, and more disease-related knowledge, avoid patients’ unrealistic expectations of treatment, and carry out more psychological care. A previous study found that shared decision-making intervention can also improve the psychological status of infertile women undergoing IVF-ET, improve their level of hope and medication adherence (Juan and Min, 2020).

This study also indicated that medication adherence was positively correlated with social support, which was consist with the results of Guo et al. (2020). A study from Kikkert Martijn et al. (2006) found that the family support with active intervention is very important for patients to get a better medication adherence. Many studies (Razali, 2010; Deane Frank et al., 2018; Stentzel et al., 2018) proved that the external support has promoting effect on medication adherence, and tried to improve patients’ medication adherence by affording more social support from various ways. This suggests that we should give more social support to the patients in clinical work. And it is recommended to try to improve their objective and subjective support and utilization of support, so that the patients can feel the care and support from their families, friends, society and medical staffs. These may reduce their mental pressure and economic pressure, and improve their medication adherence.

## Limitations

This study is subject to several limitations. First, we used a self-reported questionnaire to evaluate medication adherence, which might lead to a higher score than the actual adherence. Second, this cross-sectional study was conducted at a single center, so the generalizability of our findings may be limited. What’s more, there could still be residual variables which are not considered. Further studies should be conducted to overcome these shortcomings and consider the impact of more factors on medication adherence. In addition, the development of various strategic interventions, as well as verifications of their efficacies, is warranted.

## Conclusion

This study revealed that the medication adherence among infertile women undergoing FET cycle in China is at medium level on the whole, especially in those with repeated FET cycles. And first-time FET cycle, treatment stage, methods of daily medication, social support and hope level were the main factors associated with their medication adherence. Targeted interventions need to be developed. We recommend that efforts are focused not only in tailored approaches to health education, but also belief changes for better adherence.

## Data Availability

The raw data supporting the conclusion of this article will be made available by the authors, without undue reservation.
